# Influence of Nesquehonite on the Early-Stage Hydration of Portland Cement

**DOI:** 10.3390/ma18235271

**Published:** 2025-11-21

**Authors:** Zihan Li, Deping Chen, Teng Teng, Wenxin Liu

**Affiliations:** 1 School of Resources and Safety Engineering, University of Science and Technology Beijing, Beijing 100083, China; m202210011@xs.ustb.edu.cn (Z.L.); lwxin1999@163.com (W.L.); 2 School of Future Cities, University of Science and Technology Beijing, Beijing 100083, China; tengteng20002024@163.com

**Keywords:** nesquehonite, cement hydration, hydration product, in situ carbonation, microstructure

## Abstract

**Highlights:**

What are the main findings?

- An innovative strategy of in-situ carbon sequestration was proposed.
- For the first time, nesquehonite (NQ) was used as a CO_2_ carrier to modulate cement hydration.
- Our research elucidates the role of NQ in promoting the directed growth of hydration products in cement.

What is the implication of the main finding?

- CO_2_ and its carrier NQ serve as functional components in cement hydration.
- A quantitative relationship between NQ content and hydration product properties was established.
- A new crystal measurement method demonstrates NQ’s impact on cement hydration.

**Abstract:**

Addressing the significant pressure for carbon emission reduction in the cement industry, the development of novel cement materials capable of achieving “in situ carbon sequestration” has become an important research focus. This study introduces nesquehonite (MgCO_3_·3H_2_O, NQ) as a functional admixture into the Portland cement system, systematically investigating its effects on the cement hydration process, the evolution of hydration products, and its carbon sequestration efficiency. Through designed penetration resistance tests and hydration tests with a high water-to-solid ratio, this research utilized X-ray diffraction analysis to determine the phase composition and content of hydration products at different ages. This was combined with scanning electron microscopy to observe microstructural evolution and Nano Measure software 1.2.5 for ettringite crystal size measurement, analyzing the impact of NQ on the early hydration process of P.I cement. The results indicate that the incorporation of NQ significantly alters the early hydration of P.I cement. The Mg^2+^ and CO_3_^2−^ ions released upon its dissolution interact with Ca^2+^ and OH^−^ in the pore solution, effectively promoting the early precipitation of carbon sequestration products such as calcium carbonate and minor magnesium-containing carbonates. The addition of 10% NQ hindered the crystallization of Ca(OH)_2_ before 6 h but promoted its formation after 24 h. Mechanical property tests revealed that a sample with an optimal 3% NQ dosage not only increased the paste’s penetration resistance but also enhanced the compressive strength of the 1-day hardened sample by 8.37% compared to the plain sample, without a decrease and even a slight increase at 28 days. This enhancement is closely related to the microstructural strengthening effect induced by the carbonation products. This study confirms the feasibility of using NQ to steer the cement hydration pathway towards a low-carbon direction, revealing its dual functionality in regulating hydration and sequestering carbon within cement-based materials. The findings provide a new theoretical basis and technical pathway for developing high-performance, low-carbon cement.

## 1. Introduction

Cement, a cornerstone material in modern construction, plays an indispensable role in global industrialization and urbanization. However, the production of conventional Portland cement is associated with substantial energy consumption and greenhouse gas emissions. According to the International Energy Agency (IEA), the cement industry contributes approximately 8% of global anthropogenic CO_2_ emissions, with about 60% originating from the calcination of limestone and 40% from fuel combustion [1,2]. Against the backdrop of the global consensus on carbon neutrality goals, identifying effective strategies to reduce the carbon footprint of the cement industry has become a critical challenge for both academia and industry [3]. In recent years, decarbonizing cementitious systems through material modification, particularly by utilizing industrial by-products or functional admixtures to control the hydration process and sequester carbon dioxide, has emerged as a key research focus [4]. Within this context, nesquehonite (MgCO_3_·3H_2_O, hereafter referred to as NQ), a mineral with a unique structure and chemical activity, demonstrates significant scientific value and application potential due to its interaction with cement and its influence on the evolution of hydration products [5,6].

Cement hydration is a complex multiphase reaction process involving the dissolution, nucleation, precipitation, and microstructural evolution of primary clinker minerals such as calcium silicates and aluminates [7,8]. The type, morphology, and distribution of hydration products directly determine the mechanical properties, durability, and long-term stability of cement-based materials [9,10]. Conventional hydration products are predominantly calcium silicate hydrate (C-S-H) gel, accompanied by crystalline phases such as calcium hydroxide (Ca(OH)_2_, hereafter referred to as CH) and ettringite (3CaO·Al_2_O_3_·3CaSO_4_·32H_2_O, hereafter referred to as AFt) [11,12,13,14]. Critically, the formation of these phases does not inherently involve the utilization or sequestration of carbon dioxide. Introducing carbon-capturing components into the hydration system to shift the reaction pathway towards the formation of carbon-bearing minerals could potentially enable *in situ* carbon sequestration within the cementitious matrix.

Nesquehonite, a hydrated magnesium carbonate mineral, contains CO_3_^2−^ ions and Mg^2+^ cations within its crystalline structure. The synergistic effects of these species may profoundly influence the ionic equilibrium, nucleation kinetics, and phase assemblage of the cement paste [14]. From a thermodynamic perspective, Mg^2+^ readily combines with carbonate ions in alkaline environments to form stable magnesium carbonate hydrates. The pore solution of cement, typically highly alkaline, provides favorable conditions for such precipitation [15]. Previous studies have shown that magnesium-based compounds, such as magnesium oxide and hydroxide, can be incorporated into cement as expansive agents or reactive components to mitigate shrinkage or enhance corrosion resistance [6,14,15]. However, research on the direct interaction between nesquehonite and cement systems remains in its nascent stages. The unique layered structure and high specific surface area of nesquehonite may enable it to function as a nucleation site or a reaction template, potentially accelerating hydration reactions while promoting the formation of carbon-capturing products like calcium carbonate (CaCO_3_) or magnesium-containing carbonates [15]. These products can densify the microstructure by pore-filling [16,17,18] and, crucially, sequester CO_2_ in a stable mineral form within the cementitious matrix for long-term storage [19,20].

Current research on carbon sequestration in cement primarily focuses on techniques such as carbonation curing, pre-carbonation treatments, or the direct incorporation of carbonate minerals [21]. For instance, introducing CO_2_ during concrete mixing can enhance its utilization and reduce the carbon footprint of the industry. However, excessive CO_2_ dosing during mixing (e.g., >0.3 wt.% of cement) can adversely affect workability and compromise mechanical properties, thereby limiting its applicability [22,23,24]. In contrast, mineral carbonation (MC) has been demonstrated as a highly promising technology for CO_2_ capture and chemical sequestration [12]. Numerous studies have focused on employing magnesium-rich minerals in MC processes [25], owing to their abundant availability and the fact that the resulting carbonation products are suitable for industrial applications. Depending on the specific carbonation conditions, the primary products include magnesium carbonate (MgCO_3_) and hydrated magnesium carbonates, notably nesquehonite (NQ), dypingite (DG), and hydromagnesite (HM). Among these, NQ forms under ambient aqueous conditions at room temperature and pressure, making it the most readily obtained carbonation product [26]. Theoretically, the carbonation of 1 kg of MgO can sequester up to 1.092 kg of CO_2_, which is higher than the corresponding value of 0.785 kg for CaO. In contrast, the simultaneous promotion of hydration and carbonation through the internal incorporation of nesquehonite may offer greater uniformity and practical feasibility [27]. It is crucial to note that the introduction of magnesium ions requires careful evaluation due to potential inhibitory effects on cement hydration. Differences in ionic radius and hydration energy between Mg^2+^ and Ca^2+^ may lead to competitive adsorption on C-S-H gel surfaces, interfere with the dissolution kinetics of calcium silicates, and potentially cause delayed hydration or abnormal strength development [28]. Therefore, elucidating the dissolution-precipitation behavior of nesquehonite in cement systems, its interfacial reaction mechanisms with clinker minerals, and its consequent impact on the hydration product assemblage is essential for optimizing its application.

In summary, investigating the induced crystallization of hydration products and the carbon sequestration efficacy facilitated by nesquehonite in cement systems is of paramount importance. Such research not only deepens the understanding of cement hydration mechanisms in multi-ionic systems but also provides an innovative pathway for developing novel low-carbon cementitious materials. This study aims to systematically investigate the influence of nesquehonite incorporation on the hydration kinetics, phase composition, microstructure, and mechanical properties of cement paste using advanced microstructural characterization techniques (e.g., X-ray diffraction, scanning electron microscopy). A particular focus will be placed on the induced crystallization effects of nesquehonite on the hydration products of cement with a high water-to-cement ratio, thereby providing a new theoretical foundation and technical strategy for developing high-performance, low-carbon cement materials.

## 2. Materials and Methods

### 2.1. Raw Materials

#### 2.1.1. Portland Cement

Portland cement type P.I 42.5, conforming to the Chinese standard GB 175-2023 [29] for reference cement, was used in this study. It was obtained from Fushun Aosai’er Technology Co., Ltd., Fushun, China. This cement was selected without any supplementary cementitious materials to avoid potential influences on the hydration process. The primary clinker phases are C_3_S(Ca_3_SiO_5_), C_2_S(Ca_2_SiO_4_), C_3_A(Ca_3_(Al(OH)_6_)_2_), and C_4_AF(4CaO·Al_2_O_3_·Fe_2_O_3_). Gypsum (CaSO_4_·2H_2_O) was interground with the clinker to regulate the setting time and prevent flash setting. The chemical, physical, and mechanical properties of the cement used in the experiment are shown in Table 1, as provided by the manufacturer. The P.O cement used in this experiment was Conch brand Ordinary Portland Cement with a strength grade of 42.5, supplied by Anhui Conch Cement Company Limited, Wuhu, China. Its chemical composition and mechanical properties are shown in Table 2.

#### 2.1.2. Nesquehonite

**Figure 1:**
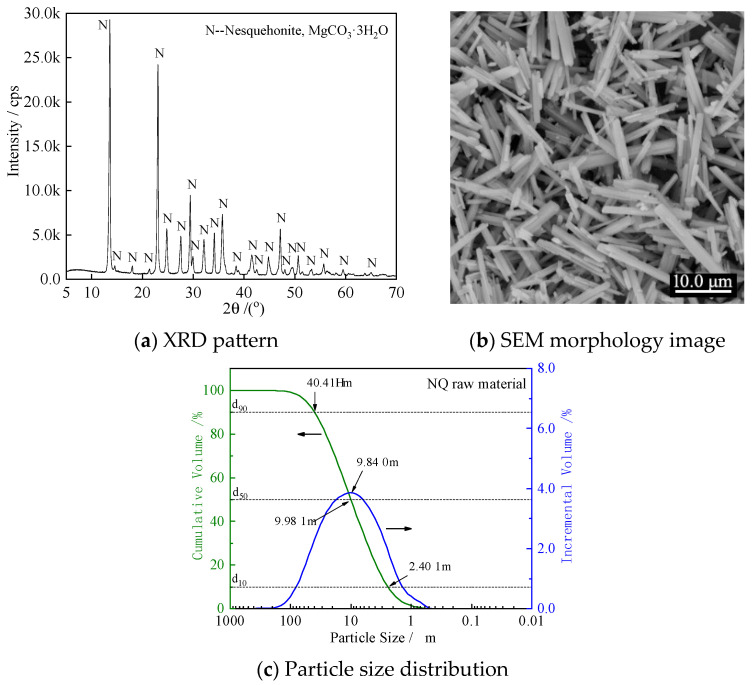
XRD pattern (**a**), SEM micrograph (**b**), and particle size distribution curve (**c**) of nesquehonite (NQ) used in the experiments.

Nesquehonite (NQ) is typically synthesized from light-burned magnesia, carbon dioxide, and water. However, residual phases such as magnesia (MgO) and brucite (Mg(OH)_2_) from the synthesis process can interfere with cement hydration. To mitigate this, high-purity nesquehonite (≥99%) supplied by Chengdu Haoming Technology Co., Ltd., Chengdu, China was used in this study. Figure 1a shows the X-ray diffraction (XRD) pattern of the nesquehonite (NQ) powder used in this study. Scanning electron microscopy (SEM) observations revealed that the NQ whiskers consist of columnar particles with varying lengths and widths, typically ranging from 5 to 40 μm in length and 0.5 to 4.0 μm in width (Figure 1b). In the alkaline environment of cement paste, NQ dissolves, releasing Mg^2+^, CO_3_^2−^, and/or HCO_3_^−^ ions, which subsequently participate in the cement hydration reactions. Laser particle size analysis indicated that the particle size distribution of the raw NQ material ranges from 0.8 to 200 μm, with d_90_, d_50_, and d_10_ values of 40.41 μm, 9.98 μm, and 2.40 μm, respectively (Figure 1c). Deionized water was used for particle-size analysis as usual, whereas tap water was employed for the other tests described in this study.

#### 2.1.3. Ground Granulated Blast Furnace Slag (GGBS)

An GGBS grade ground granulated blast furnace slag (GGBS), according to the Chinese standard GB/T 18046-2017 [30], was employed, provided by Beijing High-Strength Concrete Co., Ltd., Beijing, China. The density and specific surface area of the slag were 2.85 g/cm^3^ and 460 m^2^/kg, respectively. The main chemical composition of the slag is presented in Table 3.

**Figure 2:**
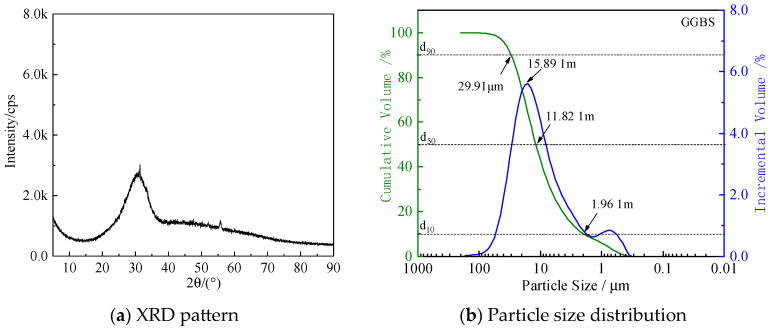
XRD pattern (**a**) and particle size distribution curve (**b**) of GGBS powder.

XRD analysis (Figure 2a) confirmed that the slag is primarily composed of amorphous phases.

Laser particle size analysis of GGBS (Figure 2b) showed that the particle sizes are predominantly distributed between 0.5 and 100 μm, with d_90_, d_50_, and d_10_ values of 29.91 μm, 11.82 μm, and 1.96 μm, respectively.

### 2.2. Experimental Methods

#### 2.2.1. Penetration Resistance Test

**Figure 3:**
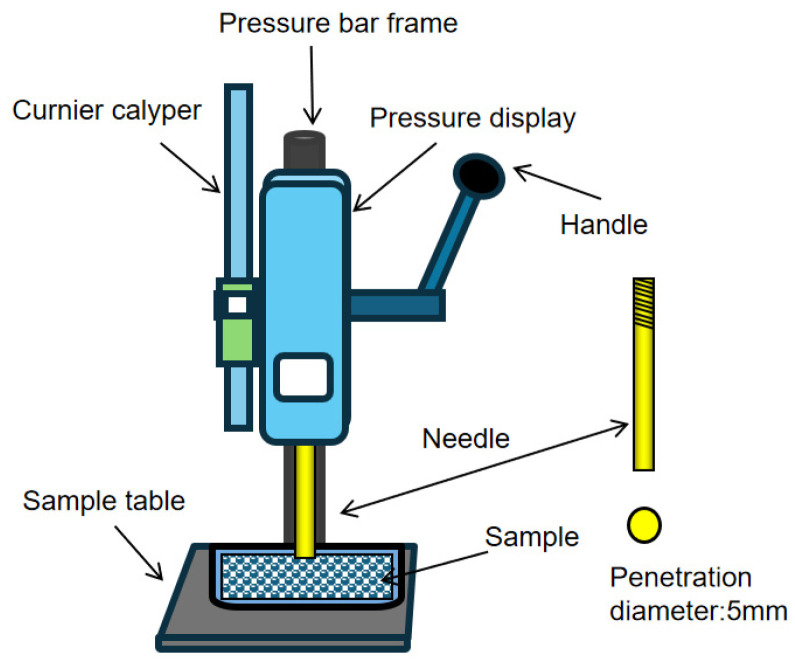
Penetration Resistance Instrument.

Portland cement was homogeneously mixed with NQ powder or NQ-slag mixtures at mass fractions of 0%, 1%, 2%, and 3%. The mixture was then mixed with water in a standard paste mixer. The resulting paste was cast into 40 mm × 40 mm × 160 mm molds, vibrated, leveled, covered with plastic film to prevent moisture loss, and cured in a standard cement curing chamber. At specified hydration ages, samples were taken for penetration resistance measurement using a penetrometer (accuracy: 1 N). The measuring instrument is shown in Figure 3. The measured force was converted into penetration resistance (stress) based on the cross-sectional area of the penetration needle. Needles with diameters of 3 mm, 4 mm, and 5 mm (corresponding cross-sectional areas: 7.07 mm^2^, 12.57 mm^2^, and 19.63 mm^2^, respectively) were available, and an appropriate needle was selected depending on the paste’s consistency. The penetration depth was fixed at 5 mm.

#### 2.2.2. Compressive Strength Test

Based on the Chinese standard GB/T 35159-2017 [31], the paste was prepared as follows: PI cement and 3% NQ were mixed in a paste mixer at 140 rpm for 10 s. After a brief pause, water was added all at once, followed by 5 s of this low-speed mixing and 15 s of high-speed mixing at 285 rpm to produce the PI–NQ paste. For comparison, a plain PI cement paste was also prepared with the same water-to-binder ratio of 0.35. The pastes were cast into 20 mm cube molds within 50 s after water addition. We prepared it with reference to Literature [32], i.e., a 20 mm × 20 mm × 20 mm cube. In our laboratory, the same dimensions were used for strength test of paste specimen in research related to nesquehonite, as shown in reference [16]. After vibration, the samples were covered with plastic film and cured in a conditioning chamber at 20 ± 2 °C and ≥95% relative humidity for 24 h before demolding. The 1-day compressive strength was then tested, while the remaining samples were cured in 20 °C water until 28 days for subsequent strength testing. Strength values represent the average of six specimens, measured using a universal testing machine. The testing machine used was a WDW-50 microcomputer-controlled electronic universal testing machine manufactured by Chaoyang Testing Instrument Co., Ltd. in Changchun, China, equipped with a DYLF series 50 kN spoke-type load cell from Bengbu Dayang Sensor System Engineering Co., Ltd. in Bengbu, China. The loading rate was 50 N/s, and the specimen was a cube with a side length of 20 mm.

#### 2.2.3. Hydration Test with High Water-to-Solid Ratio

**Figure 4:**
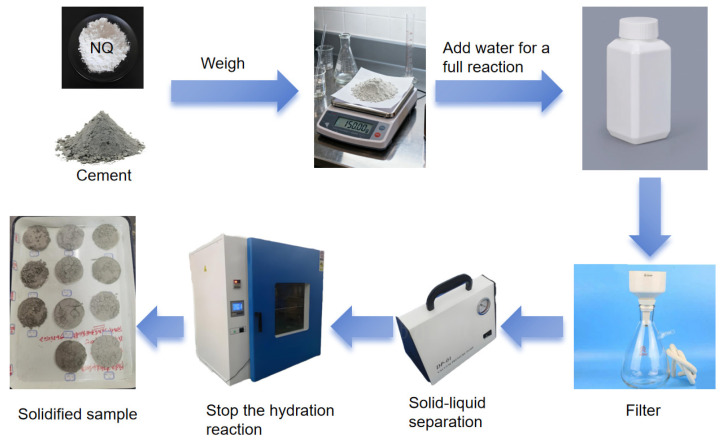
Hydration process under a high water-to-solid ratio condition.

Two batches of cement slurry were prepared by mixing P.I cement with deionized water at a water-to-cement ratio of 20:1 by mass. Separately, NQ slurries were prepared by mixing NQ powder (equivalent to 5% and 10% of the cement mass by mass, respectively) with deionized water at a water-to-NQ ratio of 20:1. The cement slurries were then mixed with the respective NQ slurries under continuous stirring. Samples of the mixed slurries were extracted at intervals of 1 min, 10 min, 1 h, 3 h, 6 h, 24 h, and 3 days after mixing. Each sample was immediately filtered. The filtrate was collected for analysis of pH value, Ca^2+^, and Mg^2+^ concentrations. The filtered solids were washed with anhydrous ethanol for moisture removal and then vacuum-dried at 40 °C ± 5 °C to stop hydration and reserved for subsequent analysis. For comparison, pure P.I cement paste and pure NQ paste samples were also prepared and sampled following the same procedure and time intervals. The complete experimental procedure is shown in Figure 4.

#### 2.2.4. Measurement of Ettringite Crystal Dimensions

**Figure 5:**
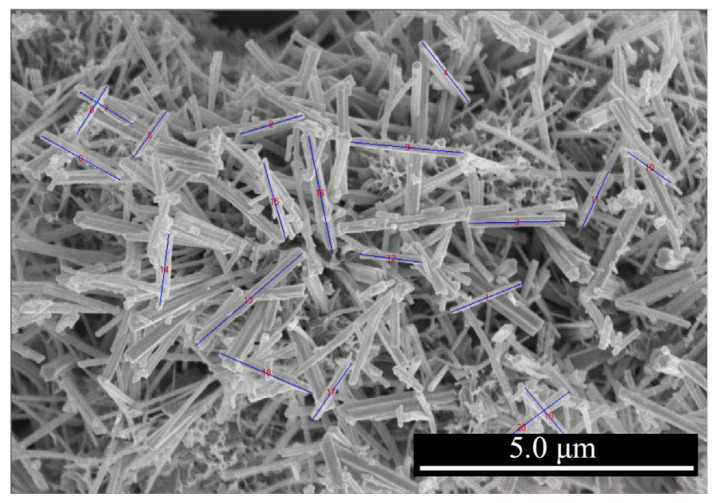
SEM image showing the morphology of ettringite crystals in Type P.I cement hydration products.

To investigate the influence of additives on the hydration process and products of P.I cement, pastes were prepared by mixing the additives with P.I cement and a large amount of water (water-to-solid ratio = 20:1). The mixed pastes were subjected to solid–liquid separation via filtration at hydration ages of 1 day and 7 days. The solid fractions were treated to stop hydration and dried at 50 °C. The morphology of the hydration products was observed using field emission scanning electron microscopy (FE-SEM). The dimensions of ettringite crystals were measured from the SEM images using Nano Measure image analysis software 1.2.5. The measurement process is shown in Figure 5.

#### 2.2.5. X-Ray Diffraction (XRD) Analysis

X-ray diffraction analysis was performed using a Bruker D8 Advance diffractometer (Bruker, Bremen, Germany) with Cu Kα radiation (λ = 1.5406 Å) operated at 40 kV. The scanning range was from 5° to 90° (2θ) with a step size of 0.02° and a scanning speed of 20°/min. A Ni filter was used to attenuate the Kβ radiation.

Prior to analysis, the samples were dried and thoroughly ground in an agate mortar until a fine, homogeneous powder was obtained.

#### 2.2.6. Scanning Electron Microscopy (SEM) Analysis

A Hitachi SU8020 cold field emission scanning electron microscope (Hitachi, Tokyo, Japan) was used for microstructural observation. This instrument utilizes the interaction between an electron beam and the specimen to generate various signals, enabling the characterization of morphology and composition. The electron gun provides resolutions of 1.0 nm (at 15 kV) and 2.0 nm (at 1 kV) for secondary electron imaging, and 3.0 nm for backscattered electron imaging. The energy dispersive X-ray spectroscopy (EDS) system can detect elements from Be to U with a resolution of 127 eV. For this study, an accelerating voltage of 5 kV was used, and the specified spot resolution was 50 nm.

For solid samples, fragments with relatively flat cross-sections (approximately 5 mm × 5 mm) were prepared, and the bottom surface was polished smooth. Powder samples were directly mounted on a metal stub using conductive adhesive. Loose particles on the surface were removed using a rubber air blower. All samples were sputter-coated with a thin layer of platinum prior to examination to enhance conductivity.

## 3. Results

### 3.1. Early-Strength Effect of NQ

**Figure 6:**
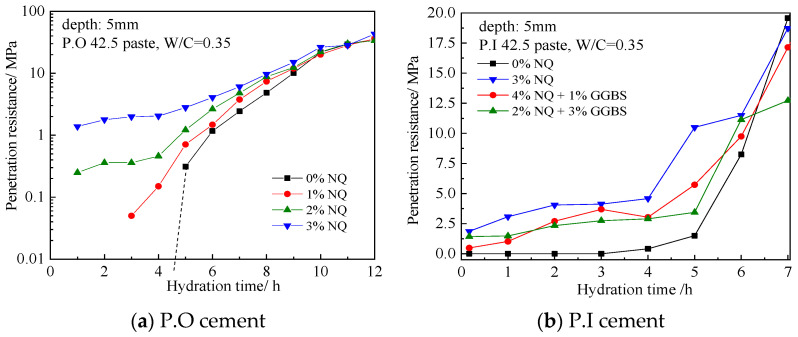
The influence of NQ addition on the variation in penetration resistance of cement slurry in the early stage of hydration.

Figure 6a shows the variation in penetration resistance with hydration time for paste samples of P.O 42.5 ordinary Portland cement incorporated with 0%, 1%, 2%, and 3% (by mass) of NQ powder. For the plain cement paste (0% NQ), a penetration resistance of 0.31 MPa was measured at 5 h, after which the value increased rapidly, reaching 21.88 MPa at 10 h. For the sample with 1% NQ, a penetration resistance of 0.05 MPa was detectable at 3 h of hydration. The samples with 2% and 3% (by mass) NQ exhibited penetration resistance values of 0.25 MPa and 1.38 MPa, respectively, at 1 h of hydration. After 10 h of hydration, the penetration resistance values of all four samples converged, ranging between 20.05 MPa and 26.42 MPa.

Considering the potential influence of the 5–20% supplementary cementitious materials in P.O ordinary Portland cement, tests were also conducted using P.I Portland cement without such materials. The effect of blending NQ with blast furnace slag (BFS) was also compared; before testing, NQ and BFS were thoroughly mixed in a high-speed mixer. The trends of penetration resistance versus hydration time for these samples are shown in Figure 6b. The sample with a blend of 4% (by mass) NQ and 1% (by mass) BFS showed lower penetration resistance within the first 5 h of hydration compared to the sample with 3% (by mass) NQ alone, but higher than the plain cement paste, and was close to the sample with a blend of 2% (by mass) NQ and 3% (by mass) BFS. The sample with 3% (by mass) NQ in P.I cement exhibited a penetration resistance of 3.08 MPa at 1 h, which is greater than the 1.38 MPa observed for the P.O cement sample with the same NQ dosage. This indicates that incorporating NQ into Portland cement can rapidly enhance paste strength. While SCMs like slag exert a slightly adverse effect on the strengthening role of NQ, this influence is not significant. To elucidate the mechanism behind the strengthening effect of NQ, subsequent experiments utilized P.I Portland cement to investigate the influence of NQ on the hydration process of clinker minerals and gypsum. The pH value was measured using an AZ8601 high-precision pH meter (AZ Instrument Corp., Taichung City, Taiwan) with a measurement range of 0–14.00 and an accuracy of ±0.02.

**Figure 7:**
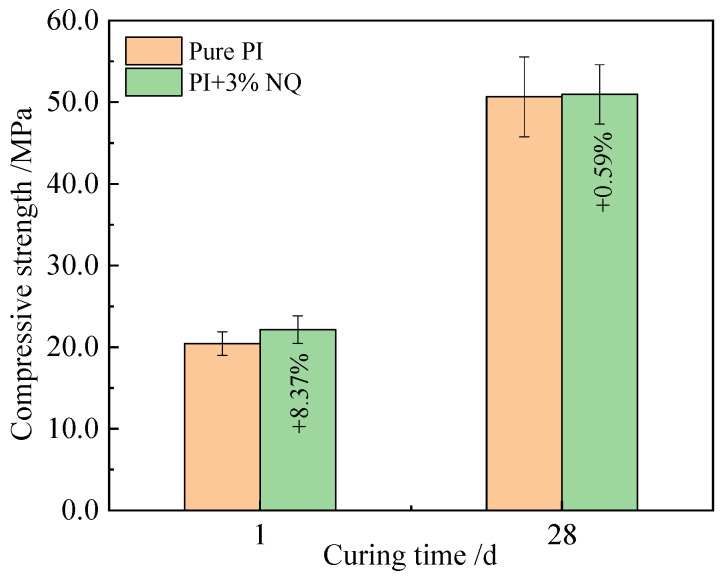
Comparison of strength between paste samples at 1d and 28d.

The compressive strengths of the plain P.I cement paste sample and the sample with 3% (by mass) NQ from Figure 6b were tested. A comparison of the results at curing ages of 1 day and 28 days is shown in Figure 7. The relevant data are presented in Table 4. Incorporating 3% (by mass) NQ not only increased the penetration resistance of the paste but also enhanced the compressive strength of the 1-day hardened sample by 8.37% compared to the plain sample. Furthermore, the strength at 28 days did not decrease but showed a slight increase.

### 3.2. Reaction Between P.I Cement and NQ Powder at High Water-to-Solid Ratio

#### 3.2.1. Analysis of Filtrate After Hydration Product Filtration

**Figure 8:**
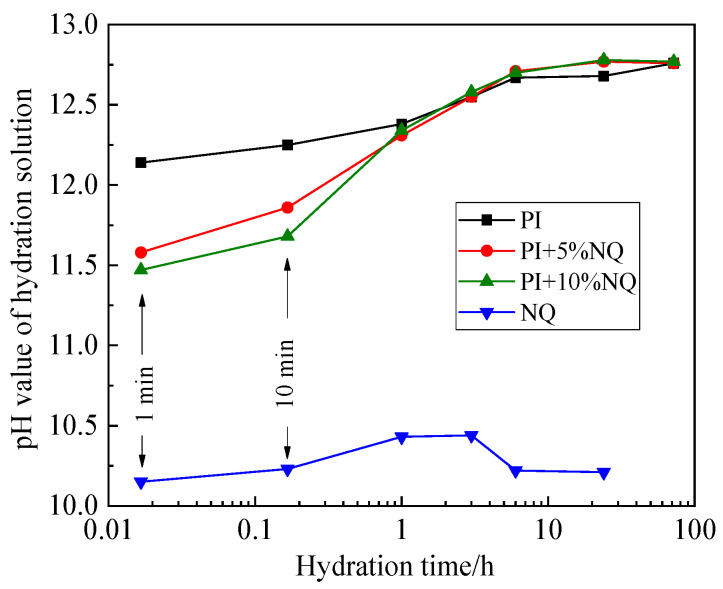
Curve of pH value of filtered solution changing with hydration time.

The variation in pH values of the filtrate measured at different hydration times is shown in Figure 8. The filtrate from the P.I cement slurry reached a pH of 12.14 just at 1 min and stabilized at 12.67 after 6 h. In contrast, the pH of the NQ slurry ranged between 10.15 and 10.44. When the NQ slurry was mixed with the cement slurry, the pH values of the filtrate at 1 min and 10 min decreased to varying degrees, only converging with the pH of the plain cement filtrate after 1 h. The decrease in filtrate pH was more pronounced for the mix with 10% (by mass) NQ than for the mix with 5% NQ.

**Figure 9:**
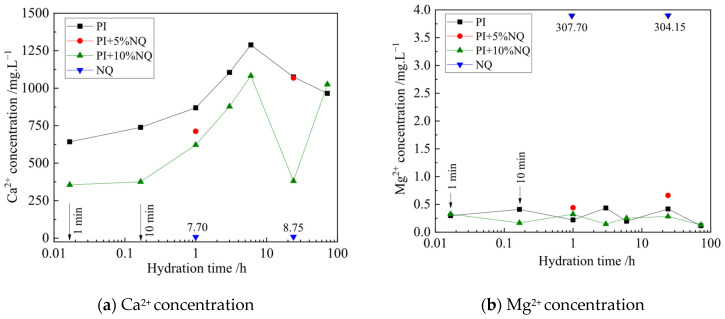
The variation curve of Ca^2+^ (**a**) and Mg^2+^ concentration (**b**) in the filtered solution with hydration.

The variations in Ca^2+^ and Mg^2+^ concentrations in the filtrate are shown in Figure 9. For the plain P.I cement slurry, the Ca^2+^ concentration in the filtrate at 1 min was 642.61 mg/L, gradually increasing with hydration time. The Ca^2+^ concentration peaked at 1288.52 mg/L in the 6 h filtrate, then decreased. The Ca^2+^ concentration in the NQ slurry filtrate was only 7.70 mg/L (1 h) and 8.75 mg/L (24 h). When 10% (by mass) NQ slurry was mixed with the cement slurry, the Ca^2+^ concentration in the 1 min filtrate dropped to 355.47 mg/L, and concentrations were lower at all times before 6 h compared to the P.I slurry. The Mg^2+^ concentrations in the filtrates of both the P.I slurry and the paste with 10% (by mass) NQ were below 0.5 mg/L. In contrast, the Mg^2+^ concentration in the NQ slurry filtrate was as high as 307.70 mg/L (1 h) and 304.15 mg/L (24 h). These results indicate that after mixing NQ into the P.I cement slurry, the Ca^2+^ concentration in the solution decreased, while the Mg^2+^ concentration did not increase. This suggests that Ca^2+^ and Mg^2+^ precipitated as insoluble compounds, removing them from the solution. The mechanism is inferred as follows: NQ dissolves in the alkaline cement solution, releasing CO_3_^2−^ and Mg^2+^ ions. These ions then react with Ca^2+^ and OH^−^ in the solution to form CaCO_3_ and Mg(OH)_2_ precipitates, leading to the observed decrease in solution Ca^2+^ concentration and pH.

#### 3.2.2. SEM Analysis of the Filtered Solid Residue

**Figure 10:**
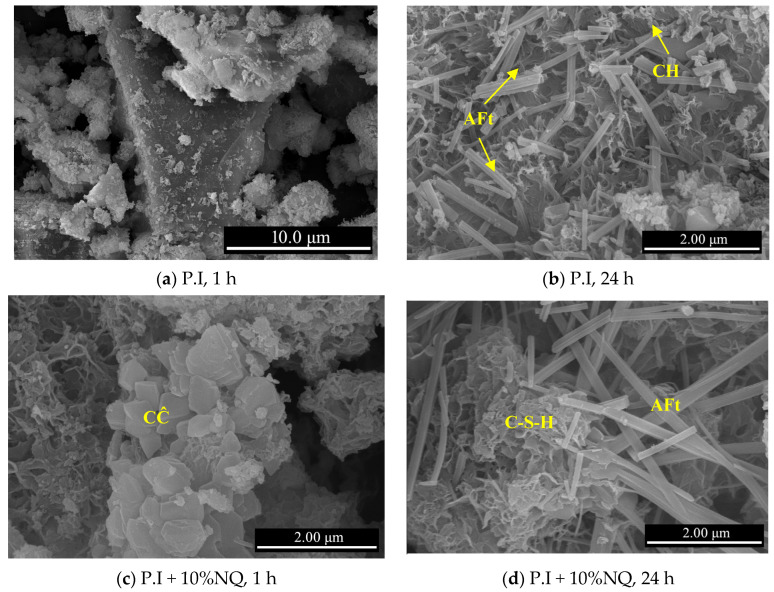
SEM micrographs of the hydration products from the plain P.I paste and the P.I paste mixed with 10% NQ.

Figure 10 shows SEM micrographs of the hydration products of the P.I slurry and its mixture with 10% (by mass) NQ at 1 h and 24 h. After 1 h of hydration of the P.I slurry, fine hydration products were observed adhering to the surfaces of the irregular cement particles (Figure 10a). At 24 h, distinct prismatic ettringite (AFt) and platy portlandite (CH) crystals were evident in the hydration products (Figure 10b). In the hydration products of the mix with 10% (by mass) NQ at 1 h, agglomerations of CaCO_3_ (CC) particles smaller than 1 μm were found (Figure 10c). At 24 h, the C-S-H gel appeared as flaky aggregates, and the AFt crystals were noticeably elongated (Figure 10d).

#### 3.2.3. XRD Analysis of the Filtered Solid Residue

(1) Key Lattice Planes and XRD Patterns

The solid residues from hydration samples at different times were analyzed by powder XRD, yielding diffraction patterns. Key lattice planes were identified and labeled on the patterns, including: the (620) plane (2θ = 51.72°) and (040) plane (2θ = 51.88°) for C_3_S; the (021) plane (2θ = 31.06°) for β-C_2_S; the (440) plane (2θ = 33.17°) for C_3_A; the (141) plane (2θ = 33.88°) for C_4_AF; the (021) plane (2θ = 20.72°) for gypsum; the (100) (2θ = 9.09°), (110) (2θ = 15.78°), and (114) (2θ = 22.94°) planes for AFt; and the (001) plane (2θ = 18.01°) for Ca(OH)_2_. Among these, the diffraction peaks for the (620) and (040) planes of C_3_S and the (021) plane of β-C_2_S do not overlap with peaks from other phases, facilitating subsequent data extraction. The (440) plane of C_3_A, the (141) plane of C_4_AF, and the (021) plane of gypsum represent their strongest diffraction peaks. The (100), (110), and (114) planes of AFt correspond to its three strongest diffraction peaks. The (001) plane of Ca(OH)_2_, representing the crystallographic direction along the thickness of the platy crystals (*Z*-axis), is its second strongest peak and is free from overlap with other phases. Standard XRD data for these key lattice planes are listed in Table 5.

**Figure 11:**
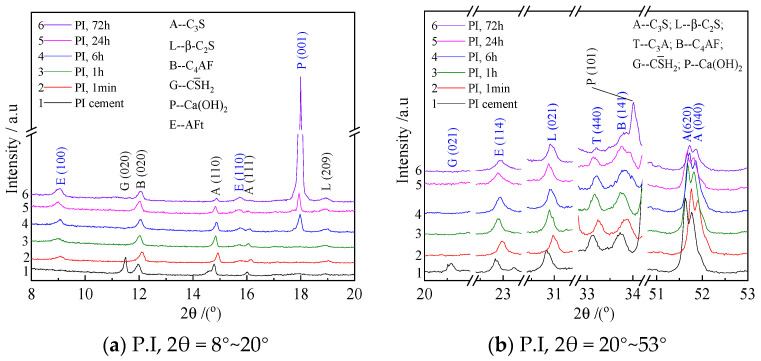
XRD patterns of P.I cement and its hydration samples.

The XRD results for the solid residues of the P.I slurry are shown in Figure 11. The key lattice planes mentioned above are annotated in blue font. The figure reveals that: (a) The diffraction peaks for the (620) and (040) planes of C_3_S, the (440) plane of C_3_A, and the (141) plane of C_4_AF are present in all samples, with their intensities decreasing to some extent as the hydration age progresses. (b) The diffraction peak intensity for the (021) plane of C_2_S shows little change within 72 h. (c) The diffraction peak for the (021) plane of gypsum disappears as early as in the 1 min hydration sample. (d) The diffraction peaks for the (100), (110), and (114) planes of AFt appear in the 1 min hydration sample, and their intensities increase with prolonged hydration age. (e) The diffraction peaks for Ca(OH)_2_ only appear in the 6 h hydration sample, and their intensity increases with age.

**Figure 12:**
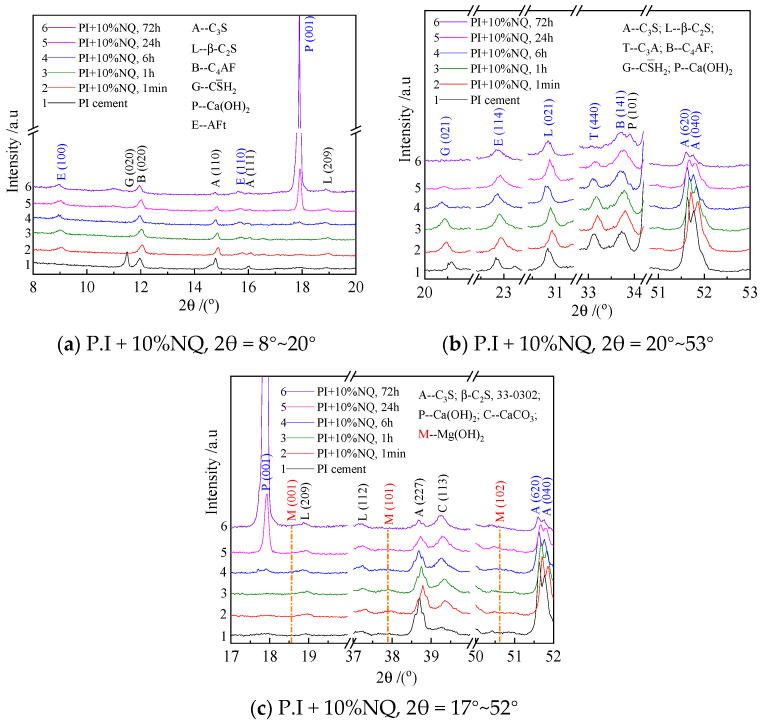
XRD patterns of hydration samples with 10% NQ added to P.I cement.

Figure 12 shows the XRD patterns of hydration samples from the P.I slurry incorporated with 10% (by mass) NQ. Comparing Figure 11 and Figure 12 reveals distinct differences: (a) Compared to the sample without NQ, the decrease in diffraction peak intensity for the (620) and (040) planes of C_3_S with hydration time is more pronounced in the sample with 10% (by mass) NQ. The diffraction peak for the (440) plane of C_3_A almost disappears in the 72 h sample. (b) The diffraction peak for the (021) plane of gypsum remains visible even in the 24 h hydration sample. (c) The addition of 10% (by mass) NQ delays the crystallization of Ca(OH)_2_; its (001) diffraction peak is not very distinct in the 6 h sample with NQ, whereas a clear peak is present in the sample without NQ at the same 6 h hydration time. It is important to note that the XRD patterns of the hydration samples with 10% (by mass) NQ do not show a diffraction peak for Mg(OH)_2_ (Figure 12c). The labeled M (101) plane in the figure corresponds to the strongest diffraction peak of Mg(OH)_2_, with a standard 2θ value of 37.983° and a d-spacing of 2.367 Å.

(2) Variation in Characteristic Peak Areas

The initial XRD data for each sample included background scattering, which affected the calculation of parameters such as peak areas for each phase. Therefore, the initial data were processed in the Jade software 6.5.26 to remove the background, followed by multi-peak fitting to extract characteristic values (including 2θ angle, d-spacing, peak intensity, full width at half maximum, and peak area) for the key lattice planes of the respective phases.

**Figure 13:**
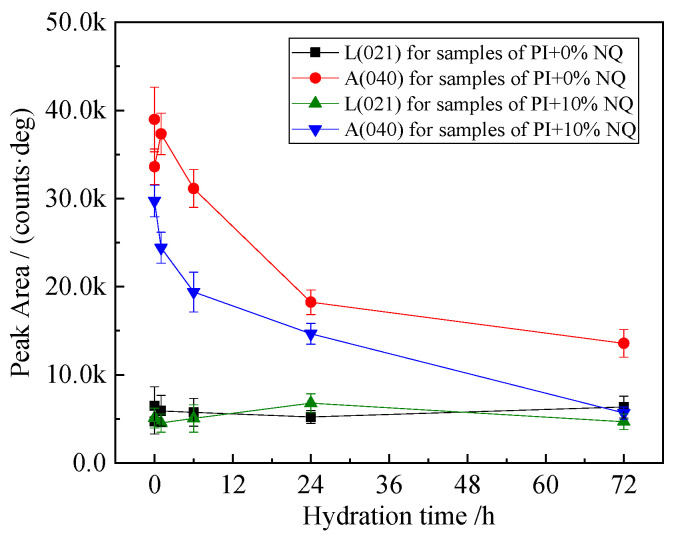
Changes in peak area of C_3_S (040) and C_2_S (021) crystal planes in the sample with hydration process.

Since the (620) and (040) planes of C_3_S are very close (differing by only 0.158° in 2θ in the standard pattern), they are collectively referred to as the (040) plane for simplicity in the following discussion. The variation in peak areas for the C_3_S (040) plane and the C_2_S (021) plane with hydration time is shown in Figure 13.

Figure 13 shows that the peak area of the C_3_S (040) plane decreases rapidly within the first 24 h of hydration. This decrease is more pronounced in the sample with 10% (by mass) NQ compared to the plain sample. In contrast, the change in the peak area of the C_2_S (021) plane is relatively minor, showing little dependence on either hydration time or the presence of NQ. This indicates that the addition of 10% (by mass) NQ has little effect on the hydration degree of C_2_S within 72 h.

(3) Variation in Relative Peak Areas of Cement Phases Normalized to C_2_S (021) Peak Area

**Figure 14:**
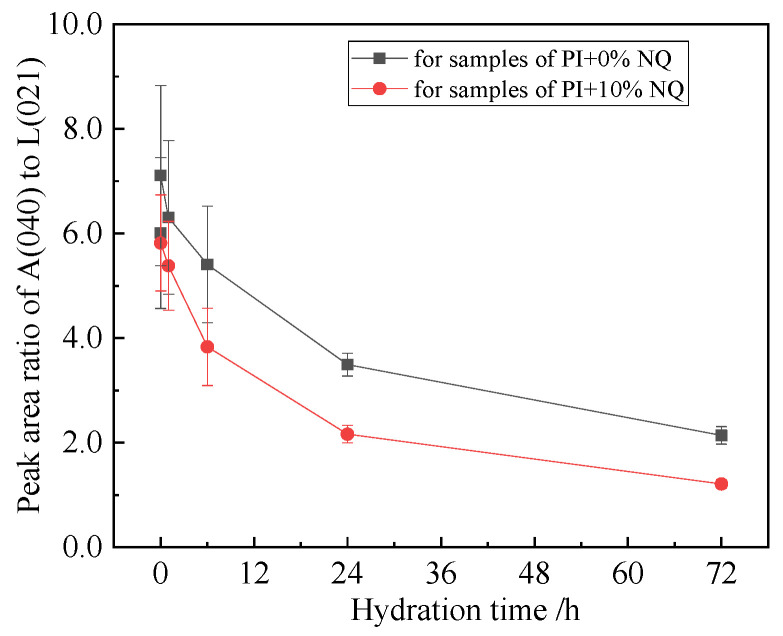
The ratio of peak area of C3S (040) crystal plane to C2S (021) crystal plane in the sample.

To account for potential errors introduced by the XRD instrument system, sample preparation, and operation across different sample batches, and considering the low hydration degree of C_2_S within 3 days and the fact that NQ addition does not promote C_2_S hydration within this period, the diffraction data were processed. Using the peak area of the C_2_S (021) plane as an internal reference, the corresponding diffraction data (e.g., peak areas) for the other phases listed in Table 3 were divided by this reference value to obtain relative peak area ratios. Figure 14 shows the variation with hydration time of the relative peak area ratio of the C_3_S (040) plane to the C_2_S (021) plane after this processing. Since peak area correlates well with phase content, subsequent analyses utilized these peak area ratios.

**Figure 15:**
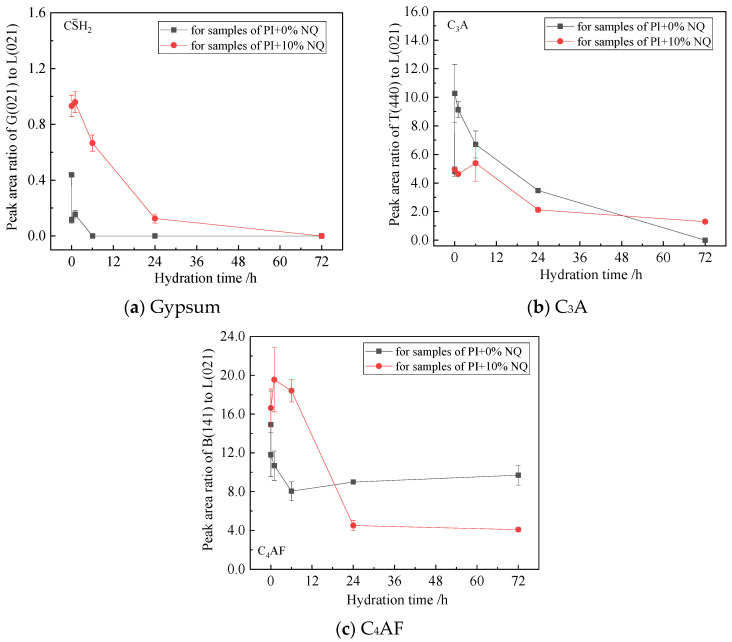
Relative peak area ratio of gypsum (021), C_3_A (440), and C_4_AF (141) crystal planes in the sample.

Figure 14 indicates that incorporating 10% (by mass) NQ additive promotes the hydration of C_3_S, correspondingly resulting in a smaller relative peak area ratio of its (040) plane to the C_2_S (021) plane. The variations in the relative peak area ratios (to C_2_S (021)) for the other three phases in P.I cement—gypsum (021), C_3_A (440), and C_4_AF (141)—with hydration time are shown in Figure 15.

For gypsum, the incorporation of 10% NQ adversely affects its dissolution within 24 h and its participation in the hydration reactions. Gypsum is not consumed rapidly and remains in the sample, manifesting as a slower decrease in the relative peak area ratio of the gypsum (021) plane with hydration time (Figure 15a). After adding 10% (by mass) NQ, the C_3_A (440) plane exhibits a lower relative peak area ratio within 24 h compared to the sample without NQ (Figure 15b), indicating that NQ promotes the hydration of C_3_A. In samples with 10% (by mass) NQ, the relative peak area ratio of the C_4_AF (141) plane is higher than in the plain sample within the first 6 h, but becomes lower after 24 h (Figure 15c), suggesting that NQ also inhibits the very early hydration of C_4_AF (before 6 h).

Analysis of the cement phases indicates that: a. NQ promotes the hydration of C_3_S but has little effect on the hydration of C_2_S within 72 h. b. NQ inhibits the early hydration of gypsum and C_4_AF but promotes the hydration of C_3_A.

(4) Variation in Relative Peak Areas of Hydration Product Phases Normalized to C_2_S (021) Peak Area

**Figure 16:**
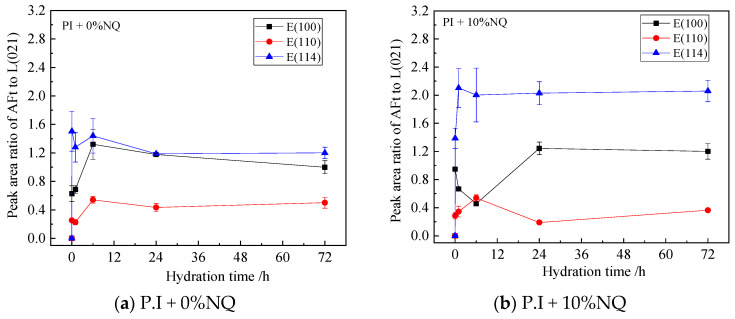
Relative peak area ratios of the three AFt lattice planes in the samples.

The variations in the relative peak area ratios (to C_2_S (021)) of the three AFt planes—(100), (110), and (114)—with hydration time are shown in Figure 16. In the sample without NQ, the peak area ratio for the (100) plane, which is the strongest diffraction peak of AFt in the standard pattern, is also relatively the highest among the three planes (Figure 16a). In the sample with 10% NQ, the peak area ratio for the (114) plane is generally higher than that for the (100) plane (Figure 16b), indicating that NQ incorporation influences the crystallization habit of AFt.

**Figure 17:**
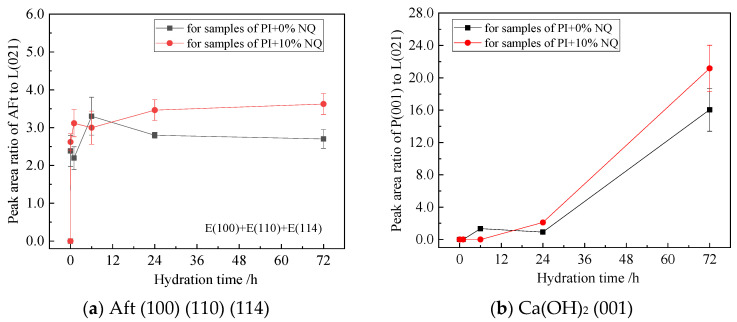
Relative peak area ratio of AFt three crystal planes and Ca(OH)_2_ (001) crystal plane in the sample.

The sum of the peak area ratios for the three AFt planes ((100), (110), (114)) versus hydration time is shown in Figure 17a. In the sample with 10% (by mass) NQ, the summed AFt peak area ratio increases rapidly with hydration time and then stabilizes, with values greater than those of the plain sample. This indicates that NQ incorporation does not hinder the rapid formation of AFt crystals and, after 24 h, may even support better crystal development. Figure 17b shows the variation in the Ca(OH)_2_ (001) plane peak area ratio with hydration time. The addition of 10% (by mass) NQ hinders the crystallization of Ca(OH)_2_ before 6 h but promotes its formation after 24 h.

**Figure 18:**
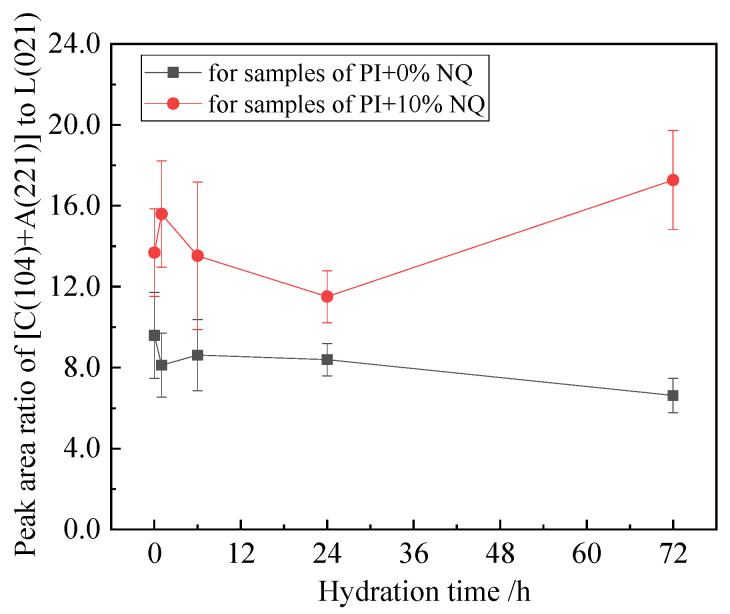
The relative peak area ratio of the overlapped peaks of the CĈ (104) crystal plane and the C_3_S (221) crystal plane in the sample.

Calcite (CaCO_3_) crystals were identified in the 1 h hydration sample with 10% (by mass) NQ. In the XRD pattern, the strongest peak of calcite CaCO_3_ is located at the (104) plane (standard 2θ = 29.405°, d = 3.035 Å), which very closely overlaps with the (221) plane of C_3_S (standard 2θ = 29.415°, d = 3.034 Å), making separation difficult. The peaks from these two planes were merged, and the ratio of this combined peak area to the C_2_S (021) peak area was plotted against hydration time, as shown in Figure 18. The curve for the sample with 10% NQ lies above that of the plain sample. Furthermore, according to Figure 14, the C_3_S peak area ratio decreases more significantly in the sample with NQ due to accelerated hydration. This confirms the precipitation of CaCO_3_ crystals upon NQ addition, occurring in large quantities within a short time (1 h).

(5) Investigation of the Effect of NQ on Ettringite Crystal Growth

Based on the aforementioned XRD analysis of hydration products and considering that the formation of AFt crystals influences the cement hydration rate, thereby regulating setting time and early strength development, the growth of ettringite crystals was statistically analyzed.

**Figure 19:**
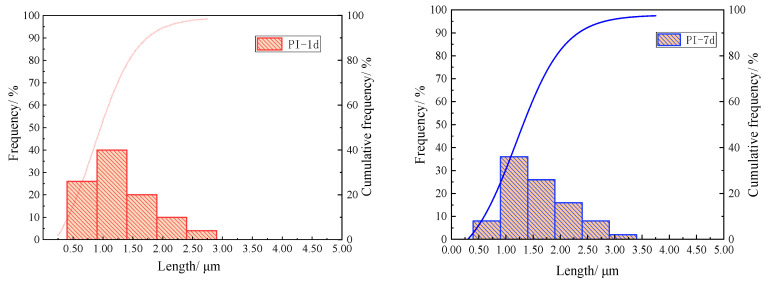

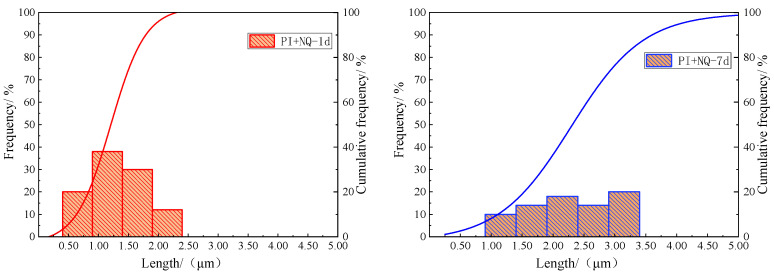
Length distribution of ettringite crystals at curing ages of 1 day and 7 days.

As shown in Figure 19, the lengths of 50 randomly selected ettringite crystals were systematically measured. The measurement data revealed the length distribution characteristics: approximately 40% of the crystals had lengths concentrated in the range of 1.50 ± 0.25 μm. In the sample with NQ, the maximum ettringite crystal length at 1 day was 2.32 μm, with an average length of 1.31 ± 0.12 μm. At 7 days, the maximum crystal length reached 5.41 μm, and the average length was 2.62 ± 0.15 μm, representing an increase of approximately 192.4% compared to the reference group. The percentages of crystals in the 2.00–2.50 μm and 3.00–3.50 μm ranges were both 20%, indicating that the incorporation of NQ led to a more uniform distribution of ettringite crystal sizes. This demonstrates that the addition of NQ effectively promotes the axial growth of ettringite crystals.

**Figure 20:**
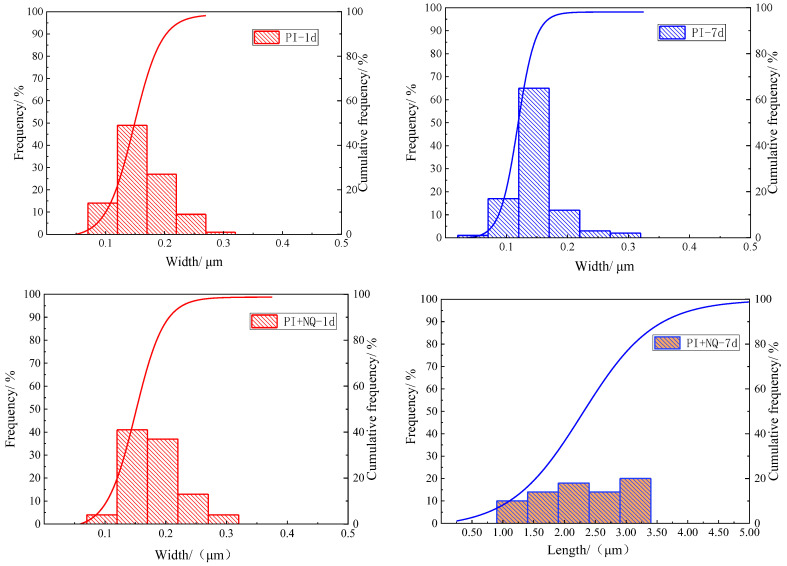
Width distribution of ettringite crystals at curing ages of 1 day and 7 days.

As shown in Figure 20, the width distribution of ettringite crystals in the cement hydration products was systematically characterized. A total of 100 representative ettringite crystals were selected for width measurement to ensure statistical reliability. The measurement results indicated that approximately 65% of the ettringite crystals had widths concentrated around 0.15 μm. This relatively narrow width distribution suggests that the nucleation and growth processes of ettringite crystals were relatively uniform under the experimental conditions. Observations at both 1 day and 7 days indicate that NQ has no significant effect on the lateral growth (width) of ettringite crystals.

## 4. Discussion

### Analysis of the Hydration Mechanism of P.I Cement with NQ

NQ dissolves in water according to Equations (1) and (2), releasing Mg^2+^, HCO_3_^−^, OH^−^, or CO_3_^2−^ ions.

MgCO_3_·3H_2_O(s) → Mg^2+^ + HCO_3_^−^ + OH^−^ + 2H_2_O (1)

MgCO_3_·3H_2_O(s) → Mg^2+^ + CO_3_^2−^ + 3 H_2_O (2)

2 (3CaO·SiO_2_) + 6H_2_O → 3CaO·2SiO_2_·3H_2_O + 3Ca^2+^ + 6OH^−^ (3)

**Figure 21:**
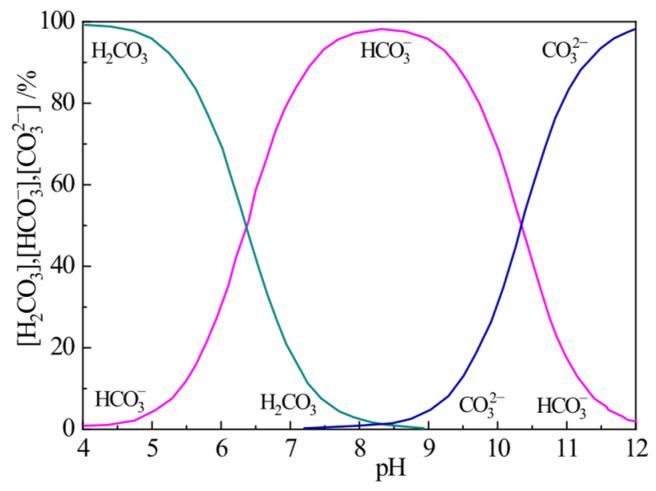
Bjerrum diagram of CO_2_-H_2_O system at 25 °C, adapted from [33].

When P.I cement is mixed with water, the clinker mineral C_3_S undergoes rapid hydration. The overall hydration process is complex, but the general reaction can be represented by Equation (3). The product 3CaO·2SiO_2_·3H_2_O is C-S-H gel, the composition of which changes during the hydration process and which exhibits poor crystallinity. As C_3_S hydrates, the concentrations of Ca^2+^ and OH^−^ in the cement paste increase rapidly until supersaturation is reached, leading to the precipitation of Ca(OH)_2_ crystals. At this point, the paste solution is saturated with Ca(OH)_2_, and the pH stabilizes at 12.67 after an initial value of 12.14, exhibiting the trend shown in Figure 8 for the plain P.I cement. According to the Bjerrum diagram for the CO_2_-H_2_O system (Figure 21) [33], when the pH exceeds 12, CO_3_^2−^ becomes the dominant carbonate species, and the HCO_3_^−^ concentration is nearly zero. Therefore, when NQ is incorporated into the P.I cement paste, it primarily dissolves according to Equation (2), producing Mg^2+^ and CO_3_^2−^ ions.

When the Ca^2+^ from the cement paste and the CO_3_^2−^ from the dissolution of NQ reach a supersaturated concentration for CaCO_3_, calcite-type CaCO_3_ precipitates, as shown in Equation (4). Consequently, CaCO_3_ crystals are observed in the hydration samples containing NQ (Figure 10c). This reaction process also leads to a decrease in the Ca^2+^ concentration in the paste (Figure 9a). The reduction in Ca^2+^ concentration, in turn, promotes the further hydration of C_3_S. Although the overall hydration of C_2_S can be represented by Equation (5), this reaction proceeds very slowly, and even with the addition of NQ, its reaction rate remains low within 72 h.

Ca^2+^ + CO_3_^2−^ → CaCO_3_(s) (4)

2 (2CaO·SiO_2_) + 4H_2_O → 3CaO·2SiO_2_·3H_2_O + Ca^2+^ + OH^−^ (5)

Mg^2+^ + 2OH^−^ → Mg(OH)_2_(s) (6)

3CaO·2SiO_2_·3H_2_O +xMg^2+^ +2xOH^−^ → 3CaO·xMgO·2SiO_2_·(3 + 2x)H_2_O (7)

The OH^−^ ions in the cement paste containing NQ react with the Mg^2+^ ions from NQ dissolution to precipitate Mg(OH)_2_ according to Equation (6). Alternatively, these ions may incorporate into the C-S-H gel structure according to Equation (7), forming a more complex C-M-S-H gel [3CaO·xMgO·2SiO_2_·(3 + 2x)H_2_O]. This leads to the observed decrease in paste pH (Figure 8) and maintains the Mg^2+^ concentration at a very low level (Figure 9b). If Mg(OH)_2_ precipitates as per Equation (6), its diffraction peaks are not detected in the samples with NQ (Figure 12c), likely due to the very poor crystallinity of the precipitated Mg(OH)_2_.

In the hydration products of cement without NQ, the gypsum (021) diffraction peak disappears quickly (Figure 11b), and its relative peak area ratio also shows a rapid decreasing trend within the first hour of hydration (Figure 15a). This corresponds to the rapid formation of AFt (Figure 17a), as gypsum reacts with C_3_A in the alkaline solution to form AFt rapidly, according to Equation (8).

3 (CaSO_4_·2H_2_O) +3CaO·Al_2_O_3_ +26H_2_O → 3CaO·Al_2_O_3_·3CaSO_4_·32H_2_O (8)

In contrast, in the hydration products with 10% NQ, the gypsum (021) diffraction peak remains visible even in the 24 h sample (Figure 12b), and the relative peak area ratio of this plane decreases slowly with hydration time (Figure 15a). However, AFt still forms rapidly in these samples (Figure 17a), and the peak area ratio of C_3_A decreases more rapidly within 24 h compared to the sample without NQ (Figure 15b). This phenomenon can be explained as follows: The CaCO_3_ generated via Equation (4) in the NQ-containing samples is highly reactive and can also rapidly react with C_3_A to form tri-carboaluminate (3CaO·Al_2_O_3_·3CaCO_3_·32H_2_O). The crystal structure of this phase is very similar to that of ettringite (3CaO·Al_2_O_3_·3CaSO_4_·32H_2_O), and both belong to the ettringite (AFt) solid solution series, as shown in Equation (9). As gypsum dissolves, SO_4_^2−^ ions are progressively incorporated into the AFt lattice, forming the AFt solid solution, as represented by Equation (10). The difference in the composition of AFt formed with and without NQ leads to slight changes in the crystal lattice, manifesting as the variations observed for the different lattice planes in Figure 16.

3CaCO_3_ + 3CaO·Al_2_O_3_ + 32H_2_O → 3CaO·Al_2_O_3_·3CaCO_3_·32H_2_O (9)

3 (CaSO_4_·2H_2_O) +3CaCO_3_ + 3CaO·Al_2_O_3_ + 26H_2_O → 3CaO·Al_2_O_3_·3(CaSO_4_,CaCO_3_)·32H_2_O (10)

In the early stages of hydration for the paste with 10% NQ, the decreased concentrations of Ca^2+^ and OH^−^ hinder the nucleation and precipitation of CH (Ca(OH)_2_). Consequently, a distinct Ca(OH)_2_ (001) diffraction peak only becomes clearly visible in the 24 h hydration products, whereas it appears in the sample without NQ as early as 6 h. Furthermore, the lower pH in the early stages of the paste with added NQ is less favorable for the formation of AFt from gypsum and C_3_A, which explains why gypsum is not consumed rapidly.

## 5. Conclusions

NQ, acting as a solid carrier of CO_2_, is highly dispersed in powder form among cement particles. It rapidly influences the properties of the entire mixing solution and induces the crystallization of cement hydration products.

(1) The incorporation of NQ has no significant effect on the lateral growth of ettringite crystals but promotes their axial growth.

(2) When introduced into a P.I cement paste, NQ rapidly decreases the paste’s pH and Ca^2+^ concentration, without increasing the Mg^2+^ concentration. This indicates the precipitation of Ca^2+^ and Mg^2+^ as insoluble compounds, removing them from the solution. SEM observations of the solid residue from the 10% (by mass) NQ sample at 1 h of hydration revealed fine agglomerations of calcite-type CaCO_3_. Furthermore, AFt crystals in the 24 h sample were noticeably longer than those in the plain sample of the same age.

(3) The incorporation of NQ into P.I cement paste promotes the hydration of C_3_S. In contrast, for C_2_S, the addition of NQ does not affect its hydration within 72 h, as evidenced by the absence of significant change in the peak area of its (021) plane with hydration time. The addition of NQ inhibits the early hydration of gypsum and C_4_AF but promotes the hydration of C_3_A.

(4) The incorporation of 10% (by mass) NQ does not hinder the rapid formation of AFt crystals. After 24 h, it even ensures better crystal development, although it does influence their crystallization habit. The addition of 10% (by mass) NQ hinders the crystallization of Ca(OH)_2_ within the first 6 h but promotes its formation after 24 h.

In subsequent studies, we will aim to achieve more precise quantification of the phase contents, thereby providing more definitive quantitative evidence for the research.

## Figures and Tables

**Table 1:** Chemical Analysis Results, Mineral Composition, and Physical Properties of P.I cement.

Chemical Composition	SiO_2_		Al_2_O_3_		Fe_2_O_3_	CaO	MgO	SO_3_			Na_2_O_eq_		f-CaO
**Fraction (%)**	20.94		4.31		3.28	63.46	2.76	2.23			0.56		0.80
**Properties**	**Fineness** **0.08/%**	**Specific surface area, m^2^/kg**		**Density,** **g/cm^3^**	**Standard consistency, %**	**Setting Time, min**		**Flexural Strength, MPa**			**Compressive Strength, MPa**		
						**Initial**	**Final**	**3 d**	**7 d**	**28 d**	**3 d**	**7 d**	**28 d**
**Measured value**	1.2	358		3.16	26.60	142	206	5.4	6.6	8.5	25.9	35.8	50.5

**Table 2:** Chemical Analysis Results, Mineral Composition, and Physical Properties of P.O cement.

Chemical Composition	SiO_2_	Al_2_O_3_	Fe_2_O_3_	CaO	MgO	SO_3_		K_2_O	LOI
**Fraction (%)**	24.25	8.15	3.14	54.22	4.90	3.74		1.31	1.42
**Properties**	**Specific surface area, m** **^2^** **/kg**	**Density,** **g/cm** **^3^**	**Initial Setting Time, min**	**Final Setting Time, min**	**Water requirement for normal consistency, g**	**Flexural** **Strength, MPa**		**Compressive Strength, MPa**	
**Measured value**	358	3.14	192	298	146	**3 d**	**28 d**	**3 d**	**28 d**
						24.5	49.2	4.3	8.5

**Table 3:** Chemical composition of GGBS for experiment.

Chemical Composition	CaO	MgO	SiO_2_	Al_2_O_3_	SO_3_	TiO	Fe_2_O_3_	Na_2_O
Content (%)	33.8	10.8	31.1	18.3	2.3	1.9	0.4	0.6

**Table 4:** Strength data of the samples at 1d and 28 d.

Sample	Curing Time	Mean/MPa	Standard Deviation/MPa
**PI**	1d	20.44	1.45
	28d	50.66	4.89
**PI + 3%NQ**	1d	22.15	1.69
	28d	50.96	3.63

**Table 5:** P.I The XRD standard diffraction angle and interplanar spacing corresponding to the main crystal planes of the P.I cement phase.

Object Phase	Phases in Type P.I Cement					Hydration Products			
	C_3_S	β-C_2_S	C_3_A	C_4_AF	Gypsum	AFt			Ca(OH)_2_
**Annotation**	A	L	T	B	G	E			P
**JCPDS#**	13-0272/ 49-0442	33- 0302	38- 1429	30- 0226	33- 0311	41-1451			44-1481
**Crystal surfacr**	(620), (040)	(021)	(440)	(141)	(021)	(100)	(110)	(114)	(001)
**2θ** **/(º)**	51.720, 51.878	31.06	33.17	33.88	20.72	9.09	15.78	22.94	18.01
**d/Å**	1.766, 1.761	2.877	2.698	2.644	4.283	9.720	5.610	3.873	4.922
**If (Strength grade number)**	40_(7)_,40_(7)_	21_(9)_	100_(1)_	100_(1)_	100_(1)_	100_(1)_	76_(2)_	31_(3)_	74_(2)_

## Data Availability

The original contributions presented in this study are included in the article. Further inquiries can be directed to the corresponding author.
