# Correction: Li et al. Influence of Nesquehonite on the Early-Stage Hydration of Portland Cement. *Materials* 2025, *18*, 5271

**DOI:** 10.3390/ma19030613

**Published:** 2026-02-05

**Authors:** Zihan Li, Deping Chen, Teng Teng, Wenxin Liu

**Affiliations:** 1 School of Resources and Safety Engineering, University of Science and Technology Beijing, Beijing 100083, China; 2 School of Future Cities, University of Science and Technology Beijing, Beijing 100083, China

In the original publication [1], there was a mistake in the citation of Figure 21. Reference [9] was incorrectly cited in Figure 21 and should be removed. In addition, “Mook, W.G. *Environmental Isotopes in the Hydrological Cycle: Principles and Applications*; IAEA: Vienna, Austria; UNESCO: Paris, France, 2000; pp. 143–165.” was not cited. The citation has now been inserted as reference [33] in Section 4, Discussion, Paragraph 2, and should read as follows: 

When P.I cement is mixed with water, the clinker mineral C_3_S undergoes rapid hydration. The overall hydration process is complex, but the general reaction can be represented by Equation (3). The product 3CaO·2SiO_2_·3H_2_O is C-S-H gel, the composition of which changes during the hydration process and which exhibits poor crystallinity. As C_3_S hydrates, the concentrations of Ca^2+^ and OH^−^ in the cement paste increase rapidly until supersaturation is reached, leading to the precipitation of Ca(OH)_2_ crystals. At this point, the paste solution is saturated with Ca(OH)_2_, and the pH stabilizes at 12.67 after an initial value of 12.14, exhibiting the trend shown in Figure 8 for the plain P.I cement. According to the Bjerrum diagram for the CO_2_-H_2_O system (Figure 21) [33], when the pH exceeds 12, CO_3_^2−^ becomes the dominant carbonate species, and the HCO_3_^−^ concentration is nearly zero. Therefore, when NQ is incorporated into the P.I cement paste, it primarily dissolves according to Equation (2), producing Mg^2+^ and CO_3_^2−^ ions.

The caption of Figure 21 has been updated accordingly and should read as follows:

**Figure 21.** Bjerrum diagram of CO_2_-H_2_O system at 25 °C, adapted from [33].

The authors state that the scientific conclusions are unaffected. This correction was approved by the Academic Editor. The original publication has also been updated.
